# Why are western diet and western lifestyle pro-inflammatory risk factors of celiac disease?

**DOI:** 10.3389/fnut.2022.1054089

**Published:** 2023-01-20

**Authors:** Kinga Skoracka, Szymon Hryhorowicz, Anna Maria Rychter, Alicja Ewa Ratajczak, Aleksandra Szymczak-Tomczak, Agnieszka Zawada, Ryszard Słomski, Agnieszka Dobrowolska, Iwona Krela-Kaźmierczak

**Affiliations:** ^1^Department of Gastroenterology, Dietetics and Internal Diseases, Poznan University of Medical Sciences, Poznań, Poland; ^2^Doctoral School, Poznan University of Medical Sciences, Poznań, Poland; ^3^Institute of Human Genetics, Polish Academy of Sciences, Poznań, Poland

**Keywords:** celiac disease, environmental factors, viruses, western diet, gut microbiota, breastfeeding, c-section, gluten

## Abstract

The prevalence of celiac disease increased in recent years. In addition to the genetic and immunological factors, it appears that environmental determinants are also involved in the pathophysiology of celiac disease. Gastrointestinal infections impact the development of celiac disease. Current research does not directly confirm the protective effect of natural childbirth and breastfeeding on celiac disease. However, it seems that in genetically predisposed children, the amount of gluten introduced into the diet may have an impact on celiac disease development. Also western lifestyle, including western dietary patterns high in fat, sugar, and gliadin, potentially may increase the risk of celiac disease due to changes in intestinal microbiota, intestinal permeability, or mucosal inflammation. Further research is needed to expand the knowledge of the relationship between environmental factors and the development of celiac disease to define evidence-based preventive interventions against the development of celiac disease. The manuscript summarizes current knowledge on factors predisposing to the development of celiac disease including factors associated with the western lifestyle.

## 1. Introduction

### 1.1. Celiac disease

Celiac disease (CD) is a systemic, T-cell mediated, autoimmune disorder that is triggered by exposure to dietary gluten in genetically predisposed individuals. This chronic disease is characterized by specific serum autoantibody response in IgG and IgA class: anti-transglutaminase IgA and anti-endomysial antibodies IgA and deamidated gliadin-related peptide IgA and IgG (1). Today, the only accepted treatment for CD is a strict gluten-free diet (2).

**Figure 1:**
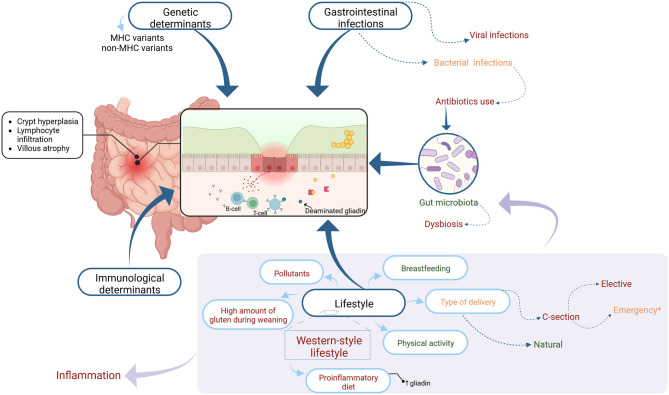
The figure shows the factors influencing and potentially influencing the development of celiac disease marked in color as appropriate: general factors-black, factors showing a potentially protective effect-green, factors negatively influencing the development of celiac disease-red, and factors that may have a dual effect-orange. Each group of factors is discussed in further detail in the text.

CD presents in about 1% of the population. Singh et al. in meta-analysis and systemic review reported that worldwide seroprevalence and prevalence of CD are 1.4 and 0.7% respectively. According to the authors, the prevalence of CD varies with sex, age, and country and it increased from 0.6% in 1991–2000 to 0.8% between 2001 and 2016 (3). It is supposed that the increase in diagnosed cases of CD is partly the result of better diagnostic tools and more frequent screening tests in individuals at risk. However, it seems that also environmental factors can contribute to that phenomenon (4). There are scientific reports, that besides genetic and immunological features, there are environmental factors that could trigger the development of CD (5). These factors (Figure 1) will be discussed in this paper.

The data presented in this study are openly available in Medline and PubMed databases and on the publisher's website. The keywords that were used for the search: celiac disease; celiac disease genetics; celiac disease risk factors; celiac disease prevention; western lifestyle; western diet; autoimmunity; inflammation; Mediterranean diet; proinflammatory diet; viruses; bacteria; infection; gut microbiota; dysbiosis; antibiotics use; breastfeeding; physical activity; c-section; gluten proteins; gluten; gliadin.

### 1.2. Gliadin and other wheat proteins

Gluten is a complex of storage proteins found in grains such as wheat, rye, and barley. In wheat, gluten constitutes mainly alcohol-soluble gliadins and insoluble glutenins. It constitutes about 85% of total grain protein mass (6). In barley, the major storage proteins are alcohol-soluble hordeins, and in the rye—secalins (7). These proteins belong to a fraction called prolamins, and they are the main endosperm storage proteins in grains (7). Wheat is the most concentrated source of gluten. The wheat kernel is one of the major cereal crops worldwide, occupying 17% of the cultivated land and constituting a staple food for 35% of the population worldwide (8). Wheat constitutes a relevant part of the contemporary diet. According to WHO, it provides 19% of the total caloric input in the population. Global wheat production amounts to an average of around 750 million tons a year and the major producers of wheat are Europe, North America, and Asia (9).

Among prolamins, the two types of proteins in comparable amounts can be distinguished: gliadins and glutenins. Gliadin, due to the high content of proline residues, is resistant to gastric-pancreatic and intestinal proteases. As result, long gliadin fragments can reach high concentrations in the gut epithelium (10). Some of the undigested peptides derived from gliadin have been reported to activate T cell response while others have pleiotropic biological activity (11). α-gliadin 33-mer peptide, P57–89 is one of the most immunogenic gluten peptides (12). It has been suggested that α-gliadin 33-mer peptide could reach lamina propria, and presented by HLA-DQ2 or 8 to T cells, can be an activator of the adaptive immune response, playing a major role in the pathogenic cascade of CD (10). Another, relatively less immunogenic peptides are 25-mer peptide, P31–43, which is not presented by HLA-DQ to T cells, or P57–73, and P111–130 (13). Undigested peptides are active *in vivo* in the celiac intestine after gluten ingestion and induce an immune response with the production of INF-alpha and IL-15 in CD biopsies and fibroblasts (14, 15).

## 2. Genetic and immunological determinants of celiac disease

Even though the inheritance of celiac disease is still unknown, it has long been known that genetics is involved in the triggering and later development of the disease (16, 17). Celiac disease, like most autoimmune diseases, is strongly associated with human leukocyte antigens (HLA) regions. The role of the HLA region as a susceptibility factor was understood in 2008 with the first genome-wide association study of celiac disease (18). However, the first reports that a major histocompatibility complex (MHC) was related to CD came 50 years earlier with the identification of the disease's association with HLA-B8 (19, 20) accounting for about 40% of CD genetic variation.

In the following years, disease associations were proven with HLA-A1, HLA-DR3, and HLA-DR7, as well as with HLA-DQ2 (21–25) which are due to the strong coupling imbalance and the fact that these alleles are part of the conservative A1-B8-DR-DQ2 haplotype.

A crucial point in the diagnosis of celiac disease was the identification of MHC class II alleles: HLA-DQ2.5, HLA-DQ8, and HLA-DQ2.2 (26). It is estimated that about 90–95% of patients with celiac disease are carriers of HLA-DQ2.5 (DQA1^*^05:01, DQB1^*^02:01). Patients who do not express HLA-DQ2.5 usually express HLA-DQ2.2 (DQA1^*^02:01, DQB1^*^02:01) or HLA-DQ8 (DQA1^*^03, DQB1^*^03:02) (27, 28). For HLA-DQ2.5, the risk of celiac disease is particularly high by the fact that carriers of the DR3DQ2 haplotype encoded by DQA1 and DQB1 have both alleles on the same chromosome-*cis* configuration, while heterozygote carriers of the DR5DQ7/DR7DQ2 haplotype have alleles on opposite chromosomes, which is *trans* configuration (28, 29).

Genome-wide association studies have so far identified 43 genetic risk factors, in addition to MHC, that explain about 50% of the genetic variation in celiac disease (17). Currently we know relatively well what role HLA-DQ2 and -DQ8 play in celiac disease (28–30) but the involvement of other loci as the celiac disease determinants, not related to HLA, is still largely undiscovered. Only three of these loci have single nucleotide polymorphisms (SNPs) located in protein-coding regions (31). Most of them—more than 90% are SNPs located in non-coding—intergenic regions, where in T and B cells they are most probably responsible for deregulating the expression of essential genes involved in the pathogenesis of celiac disease (17).

Activation of gluten-specific CD4 T lymphocytes, which recognize gluten peptides, is the first factor initiating an immune response in celiac disease. Molbergu et al. showed that gliadin-specific CD4+ T cells are present only in the mucosa of patients with celiac disease (32). This supports the concept that most T cells recognize gliadin peptide(s) when present by binding to DQ2 (33). Villi atrophy in patients after gluten ingestion can occur even after years on a gluten-free diet, proving that activation of gluten-specific T cells is crucial to the onset and pathology of celiac disease. It has also been shown that gluten-specific T cells secrete many signaling molecules upon stimulation, including interleukin IL-2, IL-4, IL-6, IL-8, IL-10, IL-21, CD40LG, IFNγ, and TNF (34–40) with IL-4, IL-21, CD40LG, and CXCL13 (C-X-C motif chemokine 13), being important in the differentiation and activation of T cells and B cells (33, 41–43). From GWAS studies, we also know that genes *SH2B3, TAGAP, PTPN2, CD28, CTLA4, CIITA*, and *IL2/IL21* are involved in T-cell activation. Interleukins *IRAK1, IL12A, IL18RAP/IL18R1, IL1RL1/IL1RL2* (44) are in turn responsible for the differentiation of inflammatory T cells CD4, while genes *IL21/IL2, RGS1, MAP3K7, CCR1,2,3, CCR4, UBASH3* are responsible for the migration of cytolytic effector T cells (44). It has also been documented that cytokines secreted by gluten-specific T cells are important for the activation and proliferation of CD8+ intraepithelial lymphocytes (IELs) in celiac disease. Thus, it follows that gluten-specific T cells play a key role in the response to gluten peptides, thereby leading to inflammation, anti-TG2 antibodies production, and atrophy of intestinal villi (45–47).

Alteration analyses of regulatory regions suggest that adaptor cells (gluten-specific T CD4 + cells and B cells) are strongly associated with celiac disease and that specific genes may contribute to the development of celiac disease through innate immune cells, epithelial cells, and D8+ TCRαβ intraepithelial cytotoxic lymphocytes (48, 49). In addition, mapping of expression quantitative trait loci (eQTL), signaling pathway analyses, and functional SNP analyses have established associations between celiac disease and T cell receptor (TCR), NFκB, and interferon (IFN) signaling pathways and several candidate genes like *UBASH3A, CD28, CSK, CD274, SH2B3*, and *STAT4* (49–51). These reports confirm the role of CD4+ T cells in celiac disease but do not, however, define how single nucleotide changes associated with CD affect gluten-specific CD4 T cells upon activation, most likely due to an incompletely understood mechanism of regulation of the stimulation response in gluten-specific T cells. In a 2018 study, Harley et al. showed that transcription factors (TFs) occupy multiple loci associated with individual complex genetic disorders, including celiac disease encompassing STAT4, STAT5A, STAT5B, T-BET, and transcription factors from the NFκB pathway. While many of these are involved in the regulation of CD4+ T-cell activation (52–55), the role of TFs as well as the transcriptional and epigenetic response in gluten-specific T cells activation has not been described, nor has the role of CD-associated genetic variants in these dynamic processes.

## 3. Infections as risk factors for celiac disease

Infections are among the environmental factors that may underline the etiopathogenesis of autoimmune diseases. The interdependencies between the influence of an infectious agent and the disease occurrence are usually complex and multidirectional. According to Lerner et al. infectious agents that could induce CD autoimmunity include Cytomegalovirus, Rotavirus, Enterovirus, Pneumococcus, *Bacteroides species, Campylobacter Jejuni, Helicobacter Pylori, Mycobacterium tuberculosis*, hepatitis B virus. Contrary to this, some studies show potential protective effects of Cytomegalovirus, *Toxoplasma gondii, H. Pylor*i, Rubella, and Epstein-Barr virus. It should be noted that the results of the research on Cytomegalovirus and *H. Pylori* infections are contradictory (56).

Jiang et al. in a meta-analysis showed that infections are associated with a 37% increase in the odds of CD, particularly among hospitalized patients. The risk was not modulated by the time of infection, type of infectious agent, and site of infection (57). Moreover, patients with CD have an increased risk of infections. Possible mechanisms for this phenomenon include hyposplenism, malnutrition, vitamin D deficiency, increased mucosal permeability, and altered immune response determined by genetic risk factors (58, 59).

Given the role of IFN secreted by host cells upon viral infection (60) in the pathophysiology of celiac disease—this cytokine has been suggested to promote T helper type 1 (Th1) responses in CD and its high levels can be observed in the duodenal mucosa from celiac patients (61)—it is speculated that viral infections are greater than bacterial risk factors for CD.

### 3.1. Viral infections

The first observations on the relationship between viral infection and CD come from the 1980s. Kagnoff et al. postulated that viruses may play a role in the pathogenesis of CD *via* an immunological cross-reaction between antigenic determinants common to the viral protein and gluten. However, this mechanism has not been confirmed (62). That observation was the beginning of further research. Beyerlein et al. analyzed data on nearly 300,000 infants from Germany. They revealed that infections of the digestive tract in the first year of life correlate with a higher risk of CD later in life (63). Also, a prospective cohort study based on The Norwegian Mother and Child Cohort Study including 72,921 children suggests that early-life infections may have a role in CD development (64).

Kemppainen et al. made similar observations after analyzing the infection data from 6,327 children from USA and Europe that carry HLA risk genotypes for CD. The researchers showed an increased risk of CD in children with recurrent infections of the digestive and respiratory tracts. The risk was modified by HLA genotype, gluten consumption, breastfeeding, and rotavirus vaccination status, and what is important, the risk was lower in children vaccinated against rotavirus (65).

Moreover, interesting are observations of Lindfors et al. who investigated the correlation between viral exposures alone or together with gluten and the risk of CD autoimmunity in 83 genetically predisposed children whose stool samples were collected monthly up to 2 years of age. The authors observed an interaction between enteroviral exposure and gluten intake suggesting that infections early in life and high gluten intake may trigger CD development in genetically predisposed children. Furthermore, they observed that children consuming bigger amounts of gluten had a higher risk of CD autoimmunity. This indicates the cumulative effect of gluten consumption and enterovirus infections in children at-risk of CD (66).

The mechanism of action of viruses in the context of gluten intolerance is not fully understood. One of the hypotheses is that gastrointestinal infections can lead to changes in the permeability of the intestinal barrier, thus leading to increased gluten penetration. The structure of the virus may be similar to gluten, which in turn may lead to an initiated anti-gluten response. Kagnoff et al. and Lähdeaho et al. in their researches demonstrated the presence of antibodies to viral peptides (62, 67). Viruses from the *Reoviridae* family are segmented, double-stranded RNA viruses (dsRNA) that cause infections in humans of all ages (68). These infections are often asymptomatic. Specific reovirus infections stimulate characteristic inflammatory pathways, thus leading to T-cell responses to food antigens, which in turn impairs the development of tolerance to the food antigen (69). Reovirus infection stimulates interferon type 1 signaling and increases the expression of the interferon regulating factor 1 (IRF1) transcription factor. Then it blocks the transition of T cells to regulatory T cells and stimulates the Th1 response to food antigens which may lead to the development of celiac disease (70, 71).

Moreover, Assa et al. observed that individuals born in May and June present a higher risk of CD (72). It may be associated with rotavirus infection, which peak occurs in early winter-the same time as exposure to gluten in children born in May and June (72). Additionally, the risk of CD is higher in children, which had a gastrointestinal infection in the first year of life (63).

However, more research is needed to fully understand the importance of viral host interactions and infection in CD development.

### 3.2. Bacterial infections and antibiotics use

Compared to the extensive, but constantly updated, knowledge of viral risk factors for CD, less is known about bacterial infections. The results of various studies on *H. pylori* infections both in children and adults are contradictory. In the meta-analysis Amlashi et al. observed a negative association between *H. pylori* and CD autoimmunity, implying a mild protective role of *H. pylori* against celiac disease (73). On the other hand, there are indications that *Campylobacter* can increase the risk of CD (74–76). The mechanism that could explain the relation between bacterial infections and CD is molecular mimicry (77).

However, antibiotics seem to increase the risk of CD through their negative effect on gut microbiota (78). Microbiome evolves during the first years of children's lives to stabilize at 3 years of age and present microbial composition similar to adult microbiota with the dominance of *Firmicutes* and *Bacteroidetes* phyla (79). Healthy gut microbiota allows for the maturation of the immune system and plays a role in the degradation of gluten in the gastrointestinal tract, affecting this way the immunogenicity of gluten peptides (78).

In meta-analysis higher doses of antibiotics correlated with a higher risk of CD. Yet, after subgroup analysis, only penicillin V was found to increase the risk of CD (57). An observational study of two nationwide cohorts including more than 1.7 million children from Denmark and Norway, among which 3,346 were diagnosed with CD, showed a positive association between antibiotic use in the first year of life with a diagnosis of CD. This relation was dose-dependent (78). Contrary to these outcomes, a systematic review analyzing data from six studies, showed no evidence of an association between prenatal or postnatal antibiotic exposure and CD (80).

## 4. Type of delivery, breastfeeding, and gluten introduction to the diet

### 4.1. Type of delivery

In the past 10 years, the global rate of cesarean section has increased to 21%, and every year it increases by 4%. In some regions of Africa, the rate of cesarean section is 4%, in Canada, the c-section rate is 27.1%, and in some countries of Latin America almost 60% (81, 82).

Children delivered by cesarean are exposed to different microbial ligands and bacterial colonization when compared to neonates born vaginally (83). Here, it is worth recalling the Danish, population-based cohort study on two million infants. The analysis showed that there is no association between cesarean delivery and CD but at the same time, cesarean delivery was positively correlated with such diseases as asthma, inflammatory bowel disease, or immune deficiencies (84). Moreover, Koletzko et al. reported a lack of association between cesarean delivery and the risk of CD or CD autoimmunity in 6,087 genetically predisposed children (85). On the other hand, a multicentre, case-control study showed, that the type of delivery may affect gut microbiota and enhance the risk of CD (86). Finally, in 2022 Yang et al. performed a meta-analysis of 11 observational studies and found that C-section is not associated with CD in offspring (87).

However, it is worth noting the study by Mårild et al. who observed that elective, but not an emergency, cesarean delivery was associated with a higher risk of CD (88). In this type of cesarean section, the birth must occur before the onset of labor thus children do not have initial contact with maternal vaginal microbiota in the birth canal. Moreover, mothers undergoing elective cesarean birth and those with an indication for scheduled deliveries are also more likely to receive antibiotic prophylaxis compared with mothers undergoing emergency C-sections. So perhaps it would be more valuable to study the impact of the antibiotic therapy used at C-section and the type of C-section rather than the impact of C-section overall on the development of CD.

### 4.2. Breastfeeding

Low- and middle-income countries generally have high rates of breastfeeding initiation of over 90%, while in high-income countries, there is a wide variation of breastfeeding initiation—countries such as Italy, Australia, or Nordic countries report rates of over 90%, while France, UK, USA, Republic of Ireland report lower rates (89). Breast milk is a complete source of nutrients for newborns, but also contains immunogenic molecules, which are immune active and affect the immune response of mucosal. Therefore, for a long time, there were suggestions, that breastfeeding may protect against CD—a meta-analyse performed in the 2006 year showed, that breastfeeding during gluten exposure reduced the risk of CD when compared with the exposition to gluten after stopping breastfeeding (90). Yet actual data do not show evidence for the preventing impact of breastfeeding. In a study performed by Lionetti et al. breastfeeding did not affect the risk of CD among children at-risk (91). Similar observations had Vriezinga et al. in the multicenter study (92). According to a systematic review by Silano et al., there is no evidence that breastfeeding duration can impact CD development in predisposed children (93). Finally, Szajewska et al. in a meta-analysis including 21 publications, showed that breastfeeding does not affect the development of CD (94).

### 4.3. Amount and time of gluten introduction to the diet

Besides, it is worth considering the quantity and timing of gluten introduction. Andrén Aronsson et al. also showed that the age of first gluten exposition is not an independent risk factor of CD (95). Similarly, in an earlier mentioned study by Lionetti et al. delayed introduction of gluten did not affect the risk of CD (91). Vriezinga et al. observed that the introduction of gluten between 16 and 24 weeks of age did not decrease the risk of CD by 3 years of age (92). In a meta-analysis performed by Szajewska et al. time of gluten introduction also did not impact CD development (94).

However, greater amounts of gluten seem to increase the risk of CD in genetically predisposed individuals. This evidence brought three separate cohort studies published in 2019 by Andrén-Aronsson et al. (96), Lund-Blix et al. (97), Mårild et al. (98). Andrén-Aronsson et al. followed for about 9 years 6,605 children. They observed that in genetically predisposed children, 20.7% of those who consumed the reference amount of gluten developed CD at 3 years. Among the group of children that consumed daily 1 g more of gluten, CD developed in 27.9% of children (96). Mårild et al. revealed that 1-year-olds with the highest gluten intake had a 2-fold greater hazard of CD, and the incidence of CD increased with higher cumulative gluten intake throughout childhood (98). Finally, Lund-Blix et al. conducted population-based research on 67,608 children independent of HLA. Increased gluten intake at 18 months was associated with a modestly increased risk of CD later in childhood. The association with gluten amount was independent of the age at the introduction of gluten. Gluten introduction ≥6 months was also an independent risk factor for CD (97). Also, the latest study by Aurrichio et al. showed that among 83 children predisposed to CD, 27 children who developed CD had eaten significantly more gluten in the second year of life than the remaining children who did not develop CD by 6 years of age (15).

Some of the studies cited in the article indicate the cumulative effects of individual factors. For example, in the study by Lindfors et al. there is an interaction between enteroviral exposures and higher gluten intake early in life indicating cumulative effects of these factors in CD development (66). Herein, we can find potential relation between increased gut permeability as an outcome of viral infection and subsequent gluten penetration. A similar relationship was observed in another incident case-referent study—it was observed that frequent infectious episodes in early life increase the risk of later celiac disease. Furthermore, there was a synergistic effect between gastrointestinal infections and the amount of daily gluten consumption, especially among infants for whom breastfeeding had been discontinued prior to gluten introduction (99).

It seems that viruses and gluten consumption may share a common pathway—a vesicular pathway regulating the innate/inflammatory response to viral ligands and bioactive dietary peptides—and trigger CD autoimmunity. The mechanism of this relation could be explained by gliadin peptide P31–43 activation of the INF alpha-mediated immune response to viruses in intestinal cells and an enterocyte cell line, CaCo-2 (14). The P31–43 peptide shows similar sequence homology with hepatocyte growth factor-regulated tyrosine kinase (HRS), which is a major regulator of endocytic vesicle maturation. Silencing or mutation of this protein contributes to delayed vesicle transport and activation of several different pathways. P31–43, therefore, delays endocytic movement in the same way as “altered” HRS leading to the same biological effects, including activation of inflammatory pathways (100).

To conclude it is worth recalling the ESPGHAN recommendations published in the 2016 year (101). Experts state that there is no evidence that neither breastfeeding nor breastfeeding during gluten introduction reduces the risk of CD. It is recommended to introduce gluten into infants' diet between 4 and 12 completed months of age—the time of gluten introduction to diet does not have an impact on overall CD development, yet the earlier introduction of gluten is associated with earlier development of CD autoimmunity. There is no recommendation regarding the type and the amount of gluten to be used at introduction but ESPGHAN suggests avoiding large quantities of gluten during the first month after gluten introduction and during infancy (101).

## 5. Western lifestyle and celiac disease

### 5.1. Western diet

**Figure 2:**
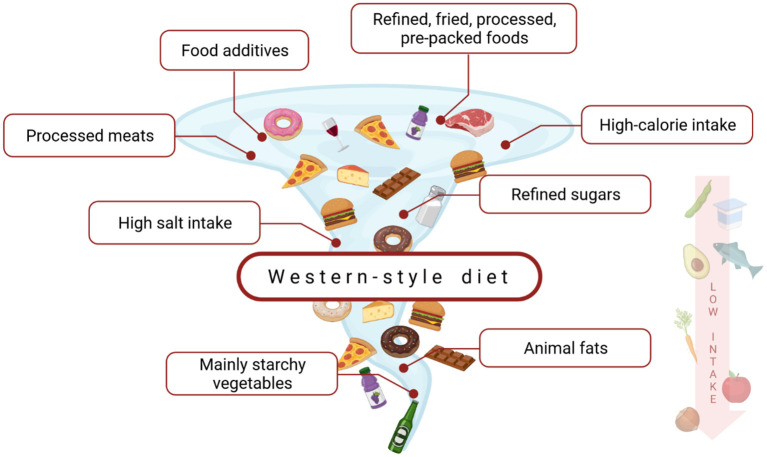
The characterization of a western-style diet. This dietary model is a high-caloric diet, rich in saturated fats, refined carbohydrates, animal protein, salt, and food additives. At the same time, it is low in fiber, vitamins, and minerals (102, 103).

Western-style diet's (WD) characteristic (Figure 2) is strongly associated with calorie-dense foods, rich in saturated fatty acids, simple carbohydrates, and animal proteins (102, 103). The term is commonly used but there is no scientifically accepted definition of WD (104). Its relationship with non-communicable diseases (NCDs), e.g., obesity or diabetes is highly discussed (105). However, there is generally little known about the impact of a western-style diet on CD. Although several causal links could be suggested, it has not been widely investigated. The possible influence of WD on CD could be associated with changes in intestinal microbiota, mucosal inflammation, and an increase in intestinal permeability further leading to bacterial translocation, endotoxemia, and systemic low-grade inflammation (106–108). WD's influence on CD seems important, as—according to the current data—its role in the pathogenesis and clinical course of other intestinal diseases, such as inflammatory bowel disease (IBD), is also suggested and investigated (109). Moreover, diet and general lifestyle are major factors of chronic NCDs and can harm immune responses and stimulate the development of a variety of inflammatory diseases (100, 110). According to the Christ et al. study, NLRP3 mediates trained immunity following a western-style diet and thus could mediate the potentially deleterious effects of trained immunity in inflammatory diseases (111).

The intestinal barrier comprises the mucus layer, intestinal epithelial cells, immune cells, gut microbiota, tight junctions located in the epithelial layer of the intestinal wall—which regulates the intestinal integrity—and gut-associated lymphoid tissue (GALT) (112). Any disruption in the components of the intestinal barrier—the physical barrier to luminal inflammatory molecules—increases its permeability, which can further implicate, e.g., inflammation in various tissues, including intestinal tissues (102, 106, 113, 114). Dietary fats expose the intestinal barrier and enhance the permeation of luminal contents into the mucosal and submucosal layers in proximity to resident immune cells, which promotes mucosal inflammation (115, 116). Moreover, high-fat diets (HFD) can enhance lipopolysaccharide (LPS) uptake and increase concentrations of bacteria-derived LPS in the circulatory system (117). Furthermore, increased permeability can also be driven by HFD-dysbiosis (118). Impaired mucosal integrity, which results in microbial translocation and increased concentrations of LPS in the bloodstream (LPS can also enter the circulation after incorporation into bile acid micelles) can lead to endotoxemia, and further low-grade systemic inflammation (119). Further, receptors such as NOD-like, C-type lectin, or toll-like receptors (TLR4) activate the NF-κB pathway increasing pro-inflammatory response and increasing concentrations of pro-inflammatory cytokines and chemokines (120). Within a short time after ingestion of saturated fats, TLR4 is downregulated and LPS translocation is increased (121).

Besides a high amount of fat, WD is also rich in simple carbohydrates, which can potentially and negatively affect intestinal permeability, possibly due to intestinal dysbiosis (122). In the study of Fajstova et al., mice fed with a high-sucrose diet had increased intestinal permeability (without damaged colon mucosa) and a higher state of immune system activation [increased proportion of neutrophils and T helper 17 cells (Th17) in the spleen, and increased proportion of Ly6C^low^, Lyc6C^high^, and macrophages in colon tissue] when compared with mice fed with control diet (120). As the authors highlighted, the negative effect of a high-sucrose diet on intestinal inflammation was not only associated with transferred microbiota but was also dependent on TLR4 signaling. Similar results were obtained by Laffin et al. where mice fed with a high-sugar diet (50% of sucrose) had increased permeability and pro-inflammatory cytokines concentrations, impaired gut microbiota, and reduced short-chain fatty acids (SCFA), and increased susceptibility to colitis (123). Frequent and high intake of animal protein is also specific for WD. However, it remains unknown how and if WD through high-protein content can affect intestinal permeability. Oddly enough, an animal study by Zhu et al. suggests that low protein intake can negatively affect the intestinal barrier (124). Possibly, animal protein can even have a positive influence on intestinal permeability, due to the high content of glutamine and tryptophan (125, 126). However, WD-derived meat is usually highly processed, and therefore, high content of saturated and trans fatty acids can shift the balance in favor of the negative influence of WD-derived meat. Further, WD is also generally low in fresh fruits and vegetables and therefore is low in anti-inflammatory and antioxidant nutrients (e.g., polyphenols, minerals, or vitamins), which additionally predisposes to low-grade chronic inflammation and possibly increased intestinal permeability (127, 128). For example, in *in vitro* study by Amasheh et al., quercetin restored TNF-α-induced intestinal permeability by downregulating claudin-2 concentrations (129, 130). A low intake of fruits and vegetables may result in a low intake of dietary fiber, which, further, predisposes to impaired intestinal barrier through reduced SCFA production and negative changes in intestinal microbiota (131).

It has been proven that anti-inflammatory diets based on the Mediterranean diet (MD) models can reduce the risk of inflammatory diseases, but still there are a lot of questions about this relation (132–134). Barroso et al. in the prospective analysis of data from the Generation R Study including 1997 children, examined associations between dietary patterns of children at 1 year with the occurrence of CD autoimmunity at 6 years. There was a correlation between the consumption of a diet that based on vegetables and grains and low amounts of refined cereals and sweet beverages, with lower odds of CD autoimmunity. These outcomes suggest that early-life dietary patterns can have an impact on CD development (135). Furthermore, Auricchio et al. conducted a study on a cohort of 239 CD genetically predisposed infants and suggested that not only the amount of gluten consumed is an important factor influencing CD development, but also dietary patterns modulate this risk. Twenty-seven children who developed CD by the age of 6 years had different dietary patterns than the remaining 56 controls, i.e., predisposed children who did not develop the disease. CD children consumed greater amounts of carbohydrates, particularly starch and sugars, and lower amounts of legumes, vegetables, fruits, and milk products. This study suggests that children who developed CD consumed a diet that was more similar to the pro-inflammatory WD than to the anti-inflammatory Mediterranean diet (15).

### 5.2. Physical activity

Important for the health of the immune system is physical activity, which may affect the production of pro-inflammatory cytokines and inflammation that underline the development of many diseases (136). The animal study showed that physical activity increased the Th17 level, which plays important role in autoimmune diseases, including CD (137, 138). Moreover, physical activity decreases the risk of autoimmune diseases. The study showed that physical activity increases Th1 production and interleukin 6 (Il-6) in muscle, which induces an anti-inflammatory response (139). However, about 31% of the worldwide population is inactive (140), which also may be a factor responsible for the increasing incidence of autoimmune diseases. Furthermore, low levels of physical activity increase the risk of being overweight and obese. The prevalence of overweight and obesity in patients with CD increases and varies from 6 to 39% and from 3 to 13%, respectively (141–145). Obesity promotes chronic, systemic low-grade inflammation and is linked to increased production of pro-inflammatory cytokines and chemokines through endocrinal active white adipose tissue (146). Considering the increasing prevalence of both CD and obesity, studies contemplating obesity as one of the risk factors for developing CD would be interesting.

### 5.3. Pollutants

Gaylord et al. analyzed persistent organic pollutant (POP) exposure in a cohort of 30 children aged 3–12 years with CD and 58 non-celiac patients aged 5–11 years presenting with gastrointestinal complaints. Authors observed the statistically significant association of POPs, especially p,p′-dichlorodiphenyldichloroethylene (DDE) with the manifestation and development of celiac disease (147). What is interesting, the meta-analysis by Wijarnpreecha et al. shows that current smokers have a significantly decreased risk of developing CD compared with never-smokers (148).

### 5.4. Gut microbiota changes

WD changes intestinal microbiota composition and increases the risk of gut dysbiosis which conversely is linked to a higher risk of CD (108, 114). The intestinal microbiota of people consuming WD is characterized by increased *Firmicutes: Bacteroidetes* ratio (105).

What is interesting, it seems that the HLA genotype impacts gut colonization (149). Herein, Olivares et al. observed in a cohort of healthy infants with at least one first-degree relative with CD, that infants carrying the HLA-DQ-2 haplotype have different microbiota composition than non-HLA-DQ-2/8-carriers. Children with a high risk of CD had increased *Firmicutes* and *Proteobacteria* proportions but reduced *Actinobacteria* proportions compared to those with low genetic risk (150). HLA-DQ-2 carriers had significantly less *Bifidobacterium* and unclassified *Bifidobacteriaceae* proportions and more *Corynebacterium, Gemella, Clostridium*, unclassified *Clostridiaceae*, unclassified *Enterobacteriaceae* and *Raoultella* proportions (150). On the other hand, gut microbiota alterations seem to contribute to the development of CD (108). In the same cohort, Olivares et al. observed that the diversity of gut microbiota in healthy children increased over time—increases in *Firmicutes* families. Those who remained healthy showed increases in TNF-α correlated to *Bifidobacterium* spp. and increased relative abundance of *Bifidobacterium longum*. Increased proportions of *Bifidobacterium breve* and *Enterococcus* spp. and reduction in secretory IgA (sIgA) levels were associated with CD development (151). Ou et al. characterized the microbiota of the small intestine in children with CD and controls. They observed that a significant fraction constituted rod-shaped bacteria—*Clostridium* spp., *Prevotella* spp., and *Actinomyces* spp.,—suggesting that it could be an important risk factor for CD, contributing to the increase in disease incidence in children below 2 years of age during Swedish CD epidemic (152).

## 6. Recommendations for the prevention of celiac disease

Actual data are insufficient to compose evidence-based recommendations for CD prevention. Yet, considering the latest findings, we can present the following directions.

First, we underline the role of the Mediterranean diet as a possible preventive approach to CD (15, 135). MD is characterized by a high intake of vegetables including legumes, olive oil, fresh, seasonal fruits, daily intake of whole grains, and regular consumption of nuts and seeds. Furthermore, according to MD's dietary pattern, fish should be consumed 2–3 times a week, dairy several times a week in moderate amounts, and meat should be consumed in moderate amounts. In addition to dishes should be used herbs and spices, and sweets should be limited. Three to four eggs per week and a moderate intake of wine can also be part of the MD diet (153). MD has the potential of a gut microbiota modulator, since consumption of MD increases the production of favorable bacteria and their metabolites, whereas dysbiosis and LPS levels decrease. In contrast to occidental dietary patterns, MD is associated with greater gut microbiota diversity and better function and integrity of the gut barrier (105).

Furthermore, taking into account the outcomes of several studies indicating the impact of viral infections on CD development, we suggest considering the preventive role of vaccination (65). Finally, it seems that in the prevention of CD bigger quantities of gluten should be avoided (101). Moreover, the latest studies indicate the cumulative effect of gluten and enterovirus infections, so it seems that especially during gastrointestinal infections gluten intake should be limited (66).

## 7. Conclusions

In conclusion, apart from genetic and immunological factors, environmental determinants play an important role in the pathogenesis of CD. Viral infections in early life seem to be important agents predisposing to CD development. On the other hand, current research does not confirm the protective effect of natural childbirth and breastfeeding on celiac disease. Regarding gluten introduction to an infant's diet—evidence shows that the time of gluten introduction does not have an impact on overall CD development but it seems that greater amounts of gluten can increase the risk of CD in genetically predisposed individuals. Furthermore, a western lifestyle, including a high-fat and high-sugar diet and a diet high in gluten, particularly gliadin, may be associated with a higher prevalence of CD. The predisposing effect of a WD on the development of CD may be related to changes in intestinal microbiota, intestinal permeability, or mucosal inflammation. Further research is needed to expand the knowledge of the relationship between environmental factors and the development of CD. Further validated studies are needed to define evidence-based preventive interventions against the development of CD.

## 8. Statement of significance

This manuscript summarizes current knowledge of the environmental factors predisposing to celiac disease development.

## Author contributions

IK-K: conceptualization. KS, SH, AMR, AER, AS-T, and AZ: writing—original draft. AMR: graphic design. IK-K, RS, AD, and KS: revision and edition of the whole manuscript. All authors have read and agreed to the present version of the manuscript.
